# Suicidality in Patients with Brain Tumors: A Brief Literature Review with Clinical Exemplar

**DOI:** 10.3390/medicina56120725

**Published:** 2020-12-21

**Authors:** Alessandra Costanza, Francesco Zenga, Roberta Rudà, Andrea Amerio, Andrea Aguglia, Gianluca Serafini, Mario Amore, Guido Bondolfi, Isabella Berardelli, Khoa Dinh Nguyen

**Affiliations:** 1 Department of Psychiatry, Faculty of Medicine, University of Geneva (UNIGE), 1211 Geneva, Switzerland; guido.bondolfi@hcuge.ch; 2 Department of Psychiatry, ASO Santi Antonio e Biagio e Cesare Arrigo Hospital, 15121 Alessandria, Italy; 3 Department of Neurosurgery, University and City of Health and Science Hospital, 10126 Torino, Italy; zengafra@hotmail.com; 4 Department of Neuro-Oncology, University and City of Health and Science Hospital, 10126 Torino, Italy; rudarob@hotmail.com; 5 Department of Neuroscience, Rehabilitation, Ophthalmology, Genetics, Maternal and Child Health, Section of Psychiatry, University of Genoa, 16133 Genoa, Italy; andrea.amerio@unige.it (A.A.); andrea.aguglia@unige.it (A.A.); gianluca.serafini@unige.it (G.S.); mario.amore@unige.it (M.A.); 6 IRCCS Ospedale Policlinico San Martino, 16133 Genoa, Italy; 7 Department of Psychiatry, Tufts University, Boston, MA 02111, USA; 8 Department of Psychiatry, Service of Liaison Psychiatry and Crisis Intervention, Geneva University Hospitals, 1211 Geneva, Switzerland; 9 Department of Neuroscience, Mental Health and Sensory Organs, Suicide Prevention Center, Sant’Andrea Hospital, Sapienza University of Rome, 00185 Rome, Italy; isabella.berardelli@uniroma1.it; 10 Department of Microbiology and Immunology, Stanford University, Palo Alto, CA 94305, USA; khoa.d.nguyen@gmail.com; 11 Tranquis Therapeutics, Palo Alto, CA 94305, USA; 12 Hong Kong University of Science and Technology, Hong Kong, China

**Keywords:** brain tumor, glioblastoma, glioma, suicide, suicidal ideation, suicide attempt, suicidal behavior, demoralization, meaning in life

## Abstract

*Background*: Suicidality and brain tumors are two life-threatening conditions and, somewhat unexpectedly, the associations between them have scarcely been reported. *Objective*: In this study, we aimed to provide a brief literature review of epidemiological studies on suicidal ideation (SI) and suicidal behavior (SB) in patients with brain tumors. To illustrate various aspects of brain tumors that potentially underlie the emergence of suicidality, the review is supplemented with a clinical exemplar of a long-term survivor of brain tumor (glioblastoma) who experienced persistent SI. Furthermore, we discuss putative both neurobiological (including anatomical and immunological) and psychosocial mechanisms that might be accountable for the development of SI and SB in patients with brain tumors. *Conclusions*: While the etiology of this phenomenon appears to be multifactorial and still remains a subject of much debate, it is of critical importance to identify patients for which a psychiatric evaluation could recognize, in a timely manner, a possible suicide risk and alleviate the deep related suffering, by appropriate psychopharmacological and supportive and psychotherapeutic interventions.

## 1. Introduction

Suicidality covers a host of phenomena, ranging from suicidal ideation (SI) to suicidal behaviors (SB), which include suicide attempts (SA), as well as completed suicide [1]. In spite of its seriousness, data on the prevalence of suicidality in individuals with a pre-existing illness [2,3,4], especially conditions with a neurological origin such as brain tumors, have not been frequently reported [5,6]. In this study, we provide a concise synthesis of the literature on this topic in order to summarize the reported frequency of SI and SB in patients with brain tumors. The review is also supplemented with a clinical case to illustrate different aspects of brain tumors that are potentially related to the development of suicidality. In the discussion, putative both neurobiological (including anatomical and immunological) and psychosocial disease mechanisms of these phenomena are highlighted [5].

## 2. Brief Literature Review

To date, few studies have investigated the association between suicidality and brain tumors (Table 1). The first incidence of suicidality in a patient with cerebral neuroblastoma was reported in 1989 [7]. Subsequently, a prospective analysis of 83 adult patients with glioma, astrocytoma, and meningioma revealed that approximately 14% exhibited SI [8], which is higher than the SI incidence in the general population (~10.8%) [9]. In contrast, Pranckeviciene et al., showed that only 5.7% of patients with high-grade glioma and meningioma reported SI [10]. These differences in suicidality rates of adult patients with brain tumors might reflect the heterogeneity of the studied participants with regard to the tumor types, as well as the suicidality assessment methods. In pediatric patients with brain tumors, the prevalence of suicidality appears to have been more thoroughly examined with the documentation of more than a 10-fold increase [11,12,13] over that of the general population (~0.6%) [14]. For instance, a study of pediatric patients with low grade glioma and embryonic tumors revealed that approximately 10.9% of patients reported SI or SA, or both [11]. Similarly, Recklitis et al., reported that 10.6% of 1136 pediatric patients exhibit symptoms of SI [12]. In another retrospective analysis of 60 pediatric patients with astrocytoma, SI was documented in 15%, while SA was present in 3.3% of the participants [13]. In spite of discrepancies in the reported rates of SI and SA, these epidemiological studies highlight the emergence of suicidality in patients with brain tumors as a clinically important phenomenon that warrants further investigations into its pathogenesis in addition to risk factors.

In this regard, the case presented below is of particular interest, due to the development of persistent SI in spite of stable neoplastic disease. Additionally, the case presents some unusual characteristics, such as the rare occurrence of a post-actinic large-vessels occlusive vasculopathy after cerebral radiotherapy (which is primarily associated with small-vessel leukoencephalopathy) and the long survival of the patient (given that the histopathological diagnosis was of glioblastoma). Both of these features will be subsequently discussed among possible mechanistic determinants that are involved in the development of SI.

## 3. Clinical Case

### 3.1. Anamnestic Elements

The patient was a married Caucasian woman who did not suffer from any major physical health problems or psychiatric illnesses (including depression or past experience of SI) before the onset of the brain tumor. She also did not have any family history of psychiatric illnesses. The patient was childless and worked as a primary school teacher. She was passionate about her work and well-respected by her colleagues. She also had a good support network of family members and friends and had no financial problems.

### 3.2. History of Present Illness

**Figure 1:**
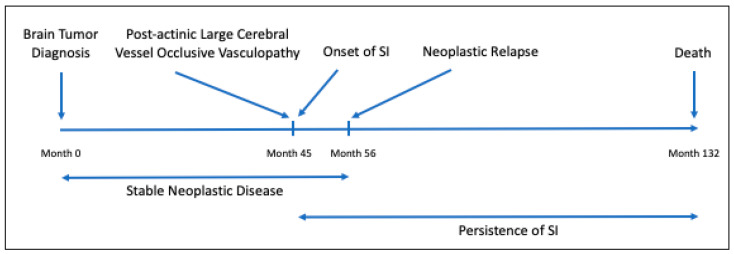
Development timeline of brain tumor and suicidal ideation (SI) in the reported clinical case. SI = suicidal ideation.

At the age of 38, the patient presented with a right partial motor seizure and secondary generalization and anomia (Figure 1). Subsequently, she underwent a macroscopically radical resection of a left frontoparietal glioblastoma, followed by external radiation with a total dose of 59.40 Gy delivered by a 6 MeV linear accelerator. This regimen consists of 45 Gy via opposed parallel fields and a booster of 14.40 Gy via isocentric oblique fields in conventional fractionation. After the completion of radiotherapy, the patient received six cycles of procarbazine, lomustine, and vincristine (PCV) chemotherapy, resulting in a partial response of 80% reduction in the tumor mass. The residual tumor remained stable for 56 months. The patient was generally in good clinical condition until the onset of chief complaints, with the exception of anterograde memory deficits that prevented her from undertaking her professional activities as a teacher.

### 3.3. Chief Complaints

Forty-five months after the completion of radiotherapy, the patient exhibited no signs of neoplastic relapse via enhanced magnetic resonance imaging (MRI). One day after this examination, the patient suddenly developed speech impairment and right brachial weakness that progressively extended to the right leg over the next three days. Concurrently, tacto-dolorific hypoesthesia and painful paresthesia developed.

This occasion marked the onset of pervasive SI. It was the first time the patient reported SI and this ideation persisted until the patient succumbed to neoplastic disease.

### 3.4. Physical and Psychiatric Examination, Laboratory Investigation and Imaging Examinations

A computerized tomography (CT) scan showed left cortical temporo-parietal hyperdensity. In the same area, an enhanced MRI revealed a widespread linear contrast enhancement along the cortical sulci (“gyral enhancement”), which indicated ischemia in the area which the middle cerebral artery divides into branches. A patient history and examinations performed during hospitalization were all negative for any general, hematological, and rheumatological risk factors for a large or medium vessel vasculopathy. Angiography did not show irregularities of the carotid and vertebra-basilar districts. Three weeks later, an MRI showed a notable tumor reduction in the pertinent areas, resulting in a nearly complete regression of the symptoms, except for mild residual aphasia. This “minor stroke” event was attributed to a radiation-induced large vessel vasculopathy due to the absence of the aforementioned risk factors and the restrictive nature of the ischemic location, which was mostly peritumoral and heavily irradiated.

Due to the onset of SI, the patient was evaluated by a psychiatrist. Diagnosis of depression was not established because of the absence of the Diagnostic and Statistical Manual of Mental Disorders; 5th edition (DSM-V) criteria. The patient was also assessed with the Montgomery–Åsberg Depression Rating Scale (MADRS) and Beck Depression Inventory-II Scale (BDI-II) [15,16] to determine the presence and severity of depression. However, the scores on both of these scales confirmed the absence of clinically relevant depression. Item 9 of the BDI-II, concerning the presence of SI, was scored “3” (“I would commit suicide if I have a chance”). The severity of SI was not assessed more precisely by a specific scale. While SI was persistent, the patient did not exhibit any SB.

### 3.5. Treatment of the Chief Complaints

Since ischemia was not considered eligible for thrombolysis, acetylsalicylic acid 100 mg/day was initiated. Even in the absence of a diagnosis of depression, the psychiatrist empirically initiated a psychoparmachological treatment (sertraline 50 mg/day with progressive increment to 100 mg/day, alprazolam 0.25 mg × 3 times a day, and olanzapine 2.5 mg/day with progressive increment to 5 mg/day) due to the presence of SI. The patient did not exhibit any psychotic symptoms. Olanzapine was introduced to potentiate the antidepressant action of sertraline, to reduce impulsivity (and possible transition to SB), and, as most suicidal patients describe, to alleviate specific deep anguish and “moral pain” associated with SI.

### 3.6. Outcome and Follow-Up

While the tumor remained stable for the next 11 months, a relapsing-remitting course of SI represented the most notable symptom even after psychopharmacological therapy, which induced no changes other than a moderate reduction in anguish and “moral pain” following the increase in olanzapine to 5 mg. Subsequently, the patient had a multicentric recurrence of the neoplastic disease and died 75 months after its incidence.

## 4. Discussion

While little is known about the etiology of suicidality in patients with brain tumors, observations from both epidemiological studies and the clinical exemplar mentioned above can provide important insights into the putative mechanisms of this phenomenon.

### 4.1. Neurobiological Risk Factors for Suicidality in Brain Tumors

The mechanistic linkage between suicidality and brain tumors could be attributed to various biological determinants of the tumors. For instance, the location of the tumors might have an important role in determining the risk for suicidality development. While suicide has been reported in sporadic cases of tumors of different nervous tissue origins [17,18,19,20], patients with lesions in the frontal lobe have a greater probability to develop SI [21,22,23,24,25]. Consistent with previous studies, the patient described in this report had a tumor in the left frontoparietal lobe, which was accompanied by SI. Such anatomical influence of the tumors might originate from the fact that their physical presence might directly impair the neuronal circuits that regulate SI and SB.

Alternatively, the histological subtype of the brain tumors might also influence the suicide risk. In support of this hypothesis, a systematic review of pediatric brain tumor survivors reported that SI was mostly associated with glioma [11,26]. Meningioma is another type of brain tumor that has been linked to increasing risk of SI development [10,27]. In line with this report, another study found that 21% of patients with benign meningioma presented with psychiatric symptoms; however, SI was not specifically investigated in these patients [7]. In spite of these findings, no clear evidence has suggested a causative association between brain tumor types and the development of suicidality. In fact, the patient in the current case and those reported by Ghaziuddin et al. [18], Nishio et al. [19], and Shehane et al. [20], had glioblastoma while others, reported by Arifin et al. [28] and Burris et al. [17], had meningioma and neurofibromatosis 1, respectively.

Other than these intrinsic determinants, the brain tumor microenvironment might also influence the development of suicidality [29,30]. In this regard, brain tumors can trigger robust local inflammatory responses [31,32]. Systemic elevation of inflammatory markers has also been linked with worsening prognosis in patients with brain tumors [33,34]. Therefore, the presence of these inflammatory mediators in the circulation and cerebrospinal fluid (CSF) might potentiate the risk for suicidality as they have been convincingly shown to be positive correlates of SI. Notably, interleukin-6 (IL-6) levels are elevated in the CSF and peripheral blood, in addition to postmortem brain specimens of suicidal patients with brain tumors [35,36,37]. Such increases in IL-6 are also associated with violent and medically serious SA or future suicide completion [38,39]. Patients with SA also exhibit high levels of other inflammatory markers, such as interferon alpha and gamma (IFN-α, -γ) and tumors necrosis factor alpha (TNF-α) [38,40]. It is worth noting that, besides these pathological inflammatory responses of the host to the tumors, brain tumor treatments, such as surgery and radiotherapy, could also elicit such events, and therefore further contribute to the risk of SI and SB in patients with brain tumors [41].

Last, but not least, the possible impact of vascular accidents induced by radiotherapy, including ischemic strokes, is of direct clinical relevance to the current case. While primarily chronic sequela resulting from cerebral radiotherapy is small-vessel leukoencephalopathy, this patient developed a rare post-actinic large-vessels occlusive vasculopathy. Corroborating studies have reported significant positive associations between stroke events and stroke-associated comorbidities and increasing risks of suicidality [42,43,44,45,46]. While the mechanisms of such events are likely to be closely related to the induction of neuroinflammatory responses during ischemic strokes [47], we did not find any evidence of neuroinflammation or systemic inflammation in the patient in this case. Such associations have not been reported in the literature, and would be of important interest for further investigations.

### 4.2. Psychosocial Risk Factors for Suicidality in Brain Tumors

In the context of nervous system tumors, the presence of psychosocial factors has been shown to be the strongest predictor of suicidality [48,49]. In this regard, evidence from this case report and previously published studies [8,9,10,11,12,13,14] suggest that SI in patients with brain tumors is linked to worsened quality of life, as the patients are constantly burdened by their unpredictable disease course and severe prognosis. Furthermore, psycho-behavioral and socioeconomic changes, such as feeling of impotence, financial distress, physical and mental disabilities, and loss of professional status, could develop. Collectively, these factors might aggravate the risk for neuropsychiatric illnesses, including suicidality. Increasing risk for the onset of SI during adulthood among pediatric survivors of brain tumors has also been reported [12,50]. Given the unexpectedly long survival of the patient affected by a glioblastoma presented in this case, the “survivor’s guilt” might have developed and ultimately triggered SI [12,50].

A number of psychological models have been investigated in our institution in the context of suicidality in patients with or without a co-morbid illness, such as the Interpersonal Theory of Suicide (IPTS) and “connectedness” [51,52,53,54,55,56,57]. Notably, the models that include “Meaning in Life” [58,59] and demoralization [60,61,62,63] constructs can be highly relevant to the phenomenon of suicidality in patients with brain tumors. According to a recent definition, “Meaning in Life” relates to one’s perception of their own coherence, purpose, and significance [64] while demoralization has historically been described as “a persistent failure to cope with stresses” [65]. The latter has five constitutive sub-components, including loss of “Meaning in Life”, hopelessness or disheartenment, helplessness, sense of failure, and dysphoria [66,67]. “Meaning in Life” and demoralization are closely and opposing linked, because meaninglessness is one of the sub-components of demoralization. These two constructs have been explored in individuals who suffer from extreme life situations, which expose them to an often incomprehensible fracture between “a before” and “an after”. In particular, they have been investigated in oncologic illnesses and palliative cares [66,67,68,69,70,71,72,73]. Notably, “Meaning in Life” and demoralization can be employed to model the development of SI and SB in non-depressed patients [66,67,74]. In patients with cancers [68,75,76,77,78] and other medical diseases [79], demoralization and depression may frequently overlap; however, the former can occur independent of the latter.

In this particular case, loss of “Meaning in Life” and demoralization may offer a non-biological explanation for the development of depression-unrelated SI, because the patient never received a formal diagnosis of depression. We postulated that her depreciated “Meaning in Life” and demoralization might result from loss of status due to cancer-induced physical and mental impairments, which are key characteristics of patients with cancer [10]. In fact, loss of status as the basis for SI development was evident in this case as the patient was professionally incapacitated due to the occurrence of language and memory deficits. Moreover, the negative impact of the disease on her status was aggravated by the fact that she was childless and her existence revolved around her work with children. This observation is consistent with the notion that the presence of children has emerged as one of the most important factors that can give a “Meaning in Life” in suicidal patients [80].

## 5. Conclusions

This succinct review highlights suicidality as a serious psychiatric condition that may accompany brain tumors. Several mechanisms could be attributed to the development of this condition, including the neuroanatomical and neuroinflammation-related intrinsic features of the tumors and how they might alter physiological pathways that are indirectly involved in the development of suicidality in addition to psychosocial stresses that can directly trigger this suicidality. While the etiology of this phenomenon appears to be multifactorial and still remains a subject of much debate, it is of critical importance to identify patients for which a psychiatric evaluation could recognize in a timely manner a possible suicide risk and alleviate the deep related suffering, by appropriate psychopharmacological and supportive and psychotherapeutic interventions [81,82].

## Figures and Tables

**Table 1:** Epidemiologic data on the occurrence of suicidal ideation and behavior (SI and SB, respectively) in patients with brain tumors.

Author	Study Type	Sample		Disease Characteristics			
		Population	Size (N)	Histological Type	Anatomical Location	Assessment	Suicidality
Pranckeviciene et al., 2016 [10]	Prospective	Adult	211	Meningioma 39% High-grade glioma 17%	-	BDI-II	5.7% of patients reported SI
Hickman et al., 2016 [8]	Prospective	Adult	83	Meningioma 32.5% Glioblastoma 28.9% Astrocytoma 7.2% Anaplastic Astrocytoma 7.2%	-	Survey	5 patients indicated SI at more than once 7 patients reported SI at least once
Brinkman et al., 2013 [11]	Retrospective	Pediatric	319	Low-grade glioma 50.8% Embryonal tumor 20.1%	Posterior fossa/cerebellum 34.5% Diencephalon/brain stem 31% Cerebral cortex 34.5%	DSM IV-TR based interview	27 patients reported SI 3 patients reported SI at more than one screening 5 patients reported SA
Recklitis et al., 2010 [12]	-	Pediatric	1136	-	-	BSI-18	10.6% of patients reported SI
Turner et al., 2009 [13]	Retrospective	Pediatric	60	Astrocytoma 33%	Posterior fossa 47% Cortical 33%	DSM IV-TR based interview	9 patients reported SI 2 patients reported SA

BDI = Beck Depression Inventory; BSI = Brief Symptom Inventory; DSM IV-TR = Diagnostic and Statistical Manual of Mental Disorders; SI = suicidal ideation; SA = suicide attempt.
